# Radiotherapy enhances natural killer cell cytotoxicity and localization in pre-clinical canine sarcomas and first-in-dog clinical trial

**DOI:** 10.1186/s40425-017-0305-7

**Published:** 2017-12-19

**Authors:** Robert J. Canter, Steven K. Grossenbacher, Jennifer A. Foltz, Ian R. Sturgill, Jiwon S. Park, Jesus I. Luna, Michael S. Kent, William T. N. Culp, Mingyi Chen, Jaime F. Modiano, Arta M. Monjazeb, Dean A. Lee, William J. Murphy

**Affiliations:** 1 Department of Surgery, Division of Surgical Oncology, University of California Davis Medical Center, Sacramento, CA 95817 USA; 2 Laboratory of Cancer Immunology, Department of Dermatology, University of California Davis Medical Center, Sacramento, CA 95817 USA; 3 Nationwide Children’s Hospital, Center for Childhood Cancer & Blood Diseases, 700 Children’s Drive, Columbus, OH 43205 USA; 4 Department of Surgery, University of California Davis Medical Center, Sacramento, CA 95817 USA; 5 The Center for Companion Animal Health, Department of Surgical and Radiological Sciences, School of Veterinary Medicine, University of California-Davis, Davis, CA 95616 USA; 6 Department of Pathology and Laboratory Medicine, UT Southwestern Medical Center, Dallas, TX 75390 USA; 7 Department of Veterinary Clinical Sciences, College of Veterinary Medicine, Animal Cancer Care and Research Center, Center for Immunology, Masonic Cancer Center, and Stem Cell Institute, University of Minnesota, St. Paul, MN 55108 USA; 8 Department of Radiation Oncology, University of California Davis Medical Center, Sacramento, CA 95817 USA; 9 Departments of Dermatology and Internal Medicine, University of California Davis Medical Center, Sacramento, CA 95817 USA; 10 Department of Surgery, Division of Surgical Oncology, UC Davis School of Medicine, 4501 X Street, Suite 3010, Sacramento, CA 95817 USA

**Keywords:** Natural killer cells, Adoptive immunotherapy, Radiotherapy, Sarcoma, Canine

## Abstract

**Background:**

We have previously shown that radiotherapy (RT) augments natural killer (NK) functions in pre-clinical models of human and mouse cancers, including sarcomas. Since dogs are an excellent outbred model for immunotherapy studies, we sought to assess RT plus local autologous NK transfer in canine sarcomas.

**Methods:**

Dog NK cells (CD5^dim^, NKp46+) were isolated from PBMCs and expanded with irradiated K562-C9-mIL21 feeder cells and 100 IU/mL recombinant human IL-2. NK homing and cytotoxicity ± RT were evaluated using canine osteosarcoma tumor lines and dog patient-derived xenografts (PDX). In a first-in-dog clinical trial for spontaneous osteosarcoma, we evaluated RT and intra-tumoral autologous NK transfer.

**Results:**

After 14 days, mean NK expansion and yield were 19.0-fold (±8.6) and 258.9(±76.1) ×10^6^ cells, respectively. Post-RT, NK cytotoxicity increased in a dose-dependent fashion in vitro reaching ~ 80% at effector:target ratios of ≥10:1 (*P* < 0.001). In dog PDX models, allogeneic NK cells were cytotoxic in ex vivo killing assays and produced significant PDX tumor growth delay (*P* < 0.01) in vivo. After focal RT and intravenous NK transfer, we also observed significantly increased NK homing to tumors in vivo. Of 10 dogs with spontaneous osteosarcoma treated with focal RT and autologous NK transfer, 5 remain metastasis-free at the 6-month primary endpoint with resolution of suspicious pulmonary nodules in one patient. We also observed increased activation of circulating NK cells after treatment and persistence of labelled NK cells in vivo*.*

**Conclusions:**

NK cell homing and cytotoxicity are increased following RT in canine models of sarcoma. Results from a first-in-dog clinical trial are promising, including possible abscopal effects.

**Electronic supplementary material:**

The online version of this article (10.1186/s40425-017-0305-7) contains supplementary material, which is available to authorized users.

## Background

In the growing field of immuno-oncology, increasing attention is being focused on the role of natural killer (NK) cells on tumor surveillance and elimination, and NK cells represent an attractive candidate for expanding the promise of immunotherapy [1–3]. To date, however, a major barrier to the successful translation of NK therapies to the clinic is that preclinical in vitro and in vivo models may not accurately reflect human spontaneous cancers where heterogeneous tumors develop over time in the setting of an intact immune system [4–6]. As a result, these “classic” pre-clinical models have been sub-optimal for assessing and optimizing key issues in NK immunotherapy, including NK homing to tumor sites, in vivo activation, and NK persistence [7, 8].

Traditionally, dogs have been used in cancer research as large animal models for safety and pharmacokinetic pre-investigational new drug studies [9, 10]. More recently, however, there is increasing focus on the investigation of companion (pet) dogs with spontaneously occurring cancers as a means to understand the biological properties and efficacy of novel drugs in settings that more closely mirror the human situation [11, 12]. For the investigation of cancer immunotherapies, dogs in particular represent an attractive addition to conventional preclinical studies. The canine genome is markedly similar to human, and dogs develop similar cancers as humans including sarcomas, melanomas, brain tumors, and lymphoma [13–15]. Importantly for the study of immunotherapy, canine cancer incidence is also associated with aging, and dog cancers develop spontaneously in the setting of an intact immune system, further underscoring the relevance of studying canine malignancies for insights into humans.

Overall, the major subsets of the dog immune system have been characterized with evidence for significant homology to humans and other mammalian species [16]. However, in contrast to other immune subsets, dog NK cells have proved more difficult to characterize as dogs do not express CD56 [5]. In fact, many of the initial descriptions of dog NK cells relied on the presence of a CD5^dim^ population in conjunction with exclusionary markers (CD3-, MHCII-, CD4-) to identify dog NK cells [17–21]. Concomitantly, similar to humans and mice, dog NK cells have also been characterized by their ability to kill tumor cells without prior antigen sensitization. Despite this indirect evidence identifying an NK-like population, a key caveat of these prior studies was whether bystander, lymphokine-activated T cells were present within the putative dog NK population. More recently, however, a canine-specific NCR-1 antibody has been shown to reliably select for canine lymphocytes lacking CD3 expression while simultaneously expressing a variety of proteins associated with both mouse and human NK cells including NKG2D, DNAM-1, NCRs, and CD16 [22–24]. Using similar protocols to isolate and expand canine natural killer cells from the peripheral blood of healthy dog donors and patients, we have observed that canine NK cells are similarly capable of killing OSA tumor cell targets in vitro and that this cytotoxicity is improved when tumor cells are pretreated with ionizing radiation (RT). We also demonstrate that adoptively-transferred canine NK cells delay the growth of canine patient-derived xenograft (PDX) tumors in vivo and that focal RT increases NK homing to canine sarcoma xenografts. Based on these findings, we initiated a first-in-dog clinical trial assessing the efficacy and tolerability of local administration of activated and expanded autologous NK cells in combination with intra-tumoral recombinant human interleukin-2 (rhIL-2) and palliative RT for dogs with spontaneous OSA. Together, our data demonstrate that dog NK cell homing and effector functions are augmented following RT in canine models of sarcoma, and results from our proof-in-concept clinical trial show the feasibility of the canine model for assessing toxicity and preliminary data for efficacy in the canine model. These data support ongoing use of the canine model for speeding the translation of novel radio-NK immunotherapy approaches to the clinic.

## Methods

### NK cell isolation and expansion

Approximately 10–15 mLs of peripheral blood was collected from healthy and tumor-bearing donors with approval from the UC Davis Institutional Animal Care and Use Committee (IACUC) and owner consent (protocol #18315). Blood was also obtained from farm-bred beagles (Ridglan Farms, Inc., Mt. Horeb, WI) using EDTA tubes diluted with sterile PBS. Lymphocytes were isolated from the buffy coat using Ficoll-Paque and dissolved in 2% canine serum containing 1 mM EDTA. PE-conjugated rat anti-canine CD5 antibody (Clone YKIX322.3, PerCP-eFluor 710, eBioscience™) was then used at a concentration of 1 μg/mL to deplete CD5^bright^ cells by negative selection with EasySep magnetic nanoparticles. The NK fraction was resuspended at 5 × 10^6^ cells/mL with irradiated K562 human feeder cells transduced with 4-1BBL (CD137L) and membrane-bound rh-IL21 (K562C9IL21, kind gift from co-investigator, DAL) at a ratio of 1:2 (NK:feeder cells) supplemented with 100 IU/mL rh-IL2 [25–28]. The parental K562 cell line was originally obtained from American Type Culture Collection (ATCC) prior to engineering of transgene expression [29]. Fresh IL-2 was added every 2–3 days while NK cells were in culture. Every 7 days, cells were counted and resuspended at a concentration 250,000 NK cells/mL with fresh K562C9IL21 at a ratio of 1:1.

### Tumor cell lines

Canine OSA tumor cell lines (OSCA-32 and -78) were obtained from established immortalized stocks (provided as kind gift from co-investigator, JFM). The NK-sensitive canine thyroid adenocarcinoma (CTAC) tumor cell line was obtained from Sigma-Aldrich® (St. Louis, MO). Cell lines were propagated in vitro with culture medium supplemented with 10% FBS, antibiotics, and L-glutamine.

### NK killing assays

For flow cytometry-based experiments, NK cytotoxicity was determined based on analysis of SSC^hi^ CD45^−^ 7AAD^−^ populations relative to control tumor cells not exposed to NK co-culture. For all in vitro RT experiments, cells were first treated with RT and then allowed to rest for 12–24 h. We also assessed NK cytotoxicity using tumor cells labelled with 100 μCi sodium chromate (^51^Cr; PerkinElmer, Boston, MA, USA) as described previously [25, 30].

### Flow cytometry and ALDEFLUOR™ assay

5 × 10^5^–10^6^ cells were stained in round bottom 96 well plates. Surface antibodies were diluted with staining buffer (2% FBS, 1 mM EDTA, and 0.02% NaN3 in PBS) and blocking buffer using canine Fc receptor binding inhibitor (ThermoFisher, #14–9162-42) and canine gamma globulin (Jackson ImmunoResearch, #004–000-002). Pacific Blue rat anti-canine CD45 (clone YKIX716.13), PerCP-eFluor™ 710 rat anti-canine CD5 (clone YKIX322.3), eFluor® 450 rat anti-canine CD8 (clone YCATE55.9) and 7-AAD were purchased from BD Biosciences (San Jose, CA). FITC mouse anti-dog CD3 (clone CA17.2A12) and AF647 mouse anti-bovine interferon-γ (clone CC302) were obtained from Bio-Rad (Hercules, CA). PE/Cy7 rat anti-canine CD4 (clone YKIX302.9) and PE/APC mouse anti-human granzyme B (clone GB12) were obtained from Thermofisher (Waltham, MA). BV711 mouse anti-human CD19 (clone HIB19) was obtained from BioLegend. For intracellular staining of interferon-γ and granzyme B, cells were mixed with viability stain, washed, and then incubated with fixation and permeabilization solution per manufacturer’s instructions (BD Biosciences). We then incubated with intracellular stain or isotype prepared in Perm/Wash Buffer™ followed by centrifugation and resuspension in 1% paraformaldehyde for flow cytometry analysis.

All data were collected using a BD Fortessa flow cytometer equipped with BD FACSDiva software. Data were analyzed using FlowJo software (TreeStar, Ashland, OR). The characteristics of our purified mouse anti-canine NKp46 antibody have been described previously. For cancer stem cell (CSC) studies, ALDEFLUOR™ expression (STEMCELL Technologies, Vancouver, BC, Canada) was determined according to the manufacturer’s instructions using diethylaminobenzaldehyde (DEAB) to inhibit aldehyde dehydrogenase (ALDH) activity and to control for background fluorescence.

### qRT-PCR

RNeasy Mini kits (Qiagen) were utilized for the extraction of total RNA from PBMCs and CD5-depleted cells. Extracted RNA was reverse transcribed to cDNA using the RNA to cDNA kit (Applied Biosystems). Gene specific primers were obtained from Integrated DNA Technologies. Quantitative real-time PCR was performed using the RT2 SYBR Green Mastermix (Qiagen) using the StepOnePlus™ Real-Time PCR system (Applied Biosystems).

### Canine PDX studies

All studies were approved by the UC Davis IACUC, and humane endpoints were used. Fresh dog sarcoma tumor specimens were obtained following either surgical resection or necropsy from the UC Davis Health Veterinary Medical Teaching Hospital (VMTH) with owner consent (protocol #18315). For PDX studies, 3–5 mm tumor fragments were implanted into the hindlimbs of female 7–8 week old NSG (NOD.Cg-Prkdcscid Il2rgtm1Wjl/SzJ) mice (Jackson Laboratories, Sacramento, CA). All mice were housed at the UC Davis animal facilities under specific pathogen-free conditions.

For in vivo therapeutic studies, mice with passage 2 sarcoma PDX tumors received either intratumoral injections 20 × 10^6^ allogeneic NK cells (from a single dog donor) with 100,000 rhIL-2 weekly × 2 or control PBS injections. Tumor volume was measured by calipers.

### Canine clinical trial

This clinical trial was approved by the UC Davis School of Veterinary Medicine Clinical Trials Review Board and IACUC (protocol #18857). Dogs were considered eligible if they were diagnosed with locally advanced, non-metastatic OSA (based on three-view thoracic radiographs), had adequate end-organ function (hematocrit >25% and renal/hepatic function within 1.5X upper limit of normal) and whose owners were not pursuing amputation and/or chemotherapy. After obtaining informed consent from owners, pre-treatment computed tomography (CT) was obtained of the primary tumor and thorax to establish a baseline for evaluating tumor response. Palliative RT was initiated within 1 week of enrollment and administered weekly at 9 Gy using 6MV photons using a clinical grade linear accelerator (TrueBeam, Varian Medical Systems, Palo Alto, CA) at the UC Davis VMTH. Prior to the last two RT sessions, peripheral blood was obtained for isolation and expansion of CD5-depleted NK cells using GMP techniques. At 1 week intervals following completion of RT, dogs received two intra-lesional injections of canine NK cells (7.5 × 10^6^ NK cells/kg) in 1–2 mL aliquots with sterile PBS. NK cells were tested prior to release to ensure that they were LPS (Limulus Amebocyte Lysate™, Lonza, Walkersville, MD) and Mycoplasma (MycoAlert™ Mycoplasma Detection Kit, Lonza) free. Prior to NK injections, dogs were pre-medicated with diphenhydramine at 2 mg/kg IM or SQ 15 min before injection. Injections were then performed under anesthesia and using either fluoroscopy or ultrasound. RhIL-2 was co-injected with NK cells at a dose of 250,000 IU/kg (Roche, NCI Frederick). We injected cells using a 20 – 22G needle, although smaller gauge needles (no smaller than 27G) were used at the discretion of the treating clinician.

The primary endpoint for this trial was toxicity of intra-tumoral autologous NK transfer following palliative RT. Adverse events were prospectively assessed using the VCOG-CTCAE v1.1 [31]. Patients were evaluated by a veterinary radiation oncologist for treatment toxicity weekly during therapy and at the 3 and 6 month follow up visits. In order to gather preliminary data for efficacy, we assessed for lung metastasis using thoracic CT every 3 months for the first 6 months of follow-up followed by Q3 month thoracic radiographs [26]. Dogs with locally advanced OSA have a poor prognosis with a risk of metastatic disease to the lungs of approximately 85% within 6 months of diagnosis. Anticipating an improvement in the rate of lung metastasis formation from the historical rate of 85% with palliative RT alone (null proportion) to 50% with palliative RT and NK transfer (alternative proportion), we determined that a trial accruing 10 dogs would have an 80% power to detect this treatment response, using a one sample binomial, a 1-sided test and a type I error rate of 5%. Based on our prior work, we hypothesized that elimination of CSCs by local injection of NK cells in the primary tumor after RT may translate to reduced metastasis formation and improved clinical outcome [25, 30].

### Recovery of NK cells after in vivo transfer

For in vivo homing studies, subcutaneous canine sarcoma PDX tumors were irradiated with 2 Gy delivered with 9 MeV electrons on an Elekta synergy clinical accelerator using a 2-cm diameter electron field and 0.5-cm bolus. Treatment accuracy was confirmed using anthropomorphic phantoms as previously described. Twelve hours later a plasmid encoding rhIL-15 was injected i.v. followed by NK transfer of 10–20 × 10^6^ ex vivo expanded allogeneic canine NK cells from a single donor. Tumors were harvested 24 h later and analyzed for mouse H2^d^−/dog CD45+/7AAD- cells by flow cytometry. In dog clinical trial patients, expanded cells were labelled using Cell Proliferation Dye eFluor® 670 prior to intra-tumoral injection. Tumor biopsy was performed 1 week later, cells were processed into single cell suspension, and flow cytometry was performed for dog CD45+/7AAD- and labelled CD45+/7AAD−/ AF647+ cells.

### Statistical analysis

Categorical variables were compared using a chi-squared test. Parametric continuous variables were compared using an independent samples t-test. Non-parametric continuous variables were compared using the Mann-Whitney U test. For comparison of more than 2 groups, statistical significance was determined using a one-way ANOVA followed by a Bonferroni multiple-group comparison test. Statistical analyses were performed using SAS version 9.2 (SAS Institute Inc., Cary, NC) and Graph-Pad Prism 5 (La Jolla, CA).

## Results

### Identification, isolation, and expansion of canine NK cells

**Fig. 1:**
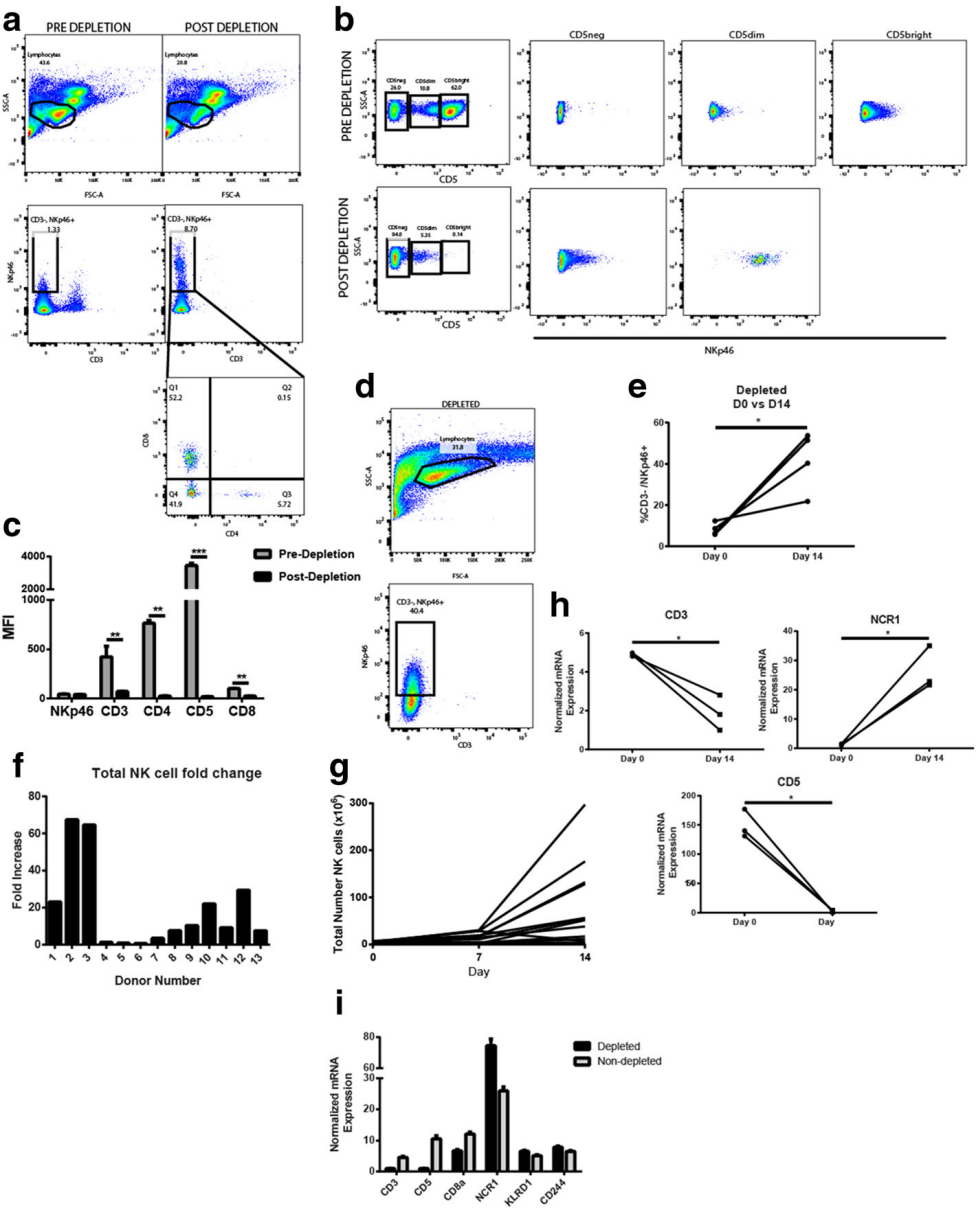
CD5 Depletion Enriches for a CD5^dim^NKp46+ Population of Dog NK Cells which Expand in Culture. **a** – **b**. Forward scatter/ side scatter was used to identify the putative lymphocyte population in dog PBMCs pre- and post-depletion of CD5^bright^ cells by negative selection using magnetic particles. Post-depletion, the proportion of CD3-NKp46+ cells is markedly increased, and these NKp46+ cells show bimodal expression of CD8 and absent expression of CD4. **c.** The lymphocyte population was analyzed for the median fluorescence intensity of indicated cell surface markers pre- and post-CD5 depletion. Post-depletion, there is significantly decreased expression of T cell surface markers as shown. **d** – **e**. At day14 after CD5-depletion and co-culture with irradiated K562 human feeder cells transduced with 4-1BBL (CD137L) and membrane-bound rh-IL21, dog NK cells demonstrate ongoing expansion of a NKp46+ population which comprise an increasing percentage of the cell product. **f**. Among 13 diverse health and tumor-bearing donors, the average NK fold expansion 14 days after isolation and co-culture was 19-fold with wide variability (SD 22.7-fold). **g.** Post-depletion, the average number of CD5^dim^ cells was 4.1 ± 1.9 × 10^6^ cells, and the average yield at day 14 was 75.2 ± 24.1 × 10^6^ cells, with greatest expansion occurring between day 7 and 14. **h** – **i**. qPCR results confirm significant down-regulation of T cell surface markers (CD3 and CD5) during co-culture from depletion to day 14 and significant up-regulation of NK cell surface marker NCR1. In each panel, a representative experiment of a minimum of 3 replicates is shown. * *P* < 0.05, ** *P* < 0.01, and *** *P* < 0.001 via paired t-test with Bonferroni correction where indicated

Given our experience with isolation, expansion, and activation of mouse and human NK cells for in vitro and in vivo studies, we first sought to evaluate optimal conditions for ex vivo culture of canine NK cells. Prior investigators have identified a CD5^dim^/CD3variable/CD8+/TCRαβ/γδ- putative canine NK population which is responsive to human cytokine stimulation as well as co-culture with an immortalized mouse embryonic feeder cell line (EL08-1D2) and a lethally irradiated immortalized human myelogenous leukemia line expressing co-stimulatory transgenes. Based on these descriptions, we utilized a similar CD5 depletion strategy to deplete CD5^high^ cells (Fig. 1a-b) from PBMCs obtained from multiple donors including healthy dog volunteers, tumor-bearing dogs, and laboratory beagles. CD5 depletion lead to a significant reduction in CD5^high^ and CD3+ cells from canine PBMCs, leaving both CD5^dim^ and CD3- populations (Fig. 1a-b). Importantly, we observed a significant enrichment in NKp46+/CD3- cells in the CD5^dim^ population (Fig. 1a, b, d, e). Depletion was also effective at removing CD4+ and CD8+ cells, although a significant CD8+ population remained and expanded (Fig. 1c, i).

Following CD5 depletion, the remaining cell population was cultured in the presence of an irradiated human feeder cell line (K562C9IL21), which we and others have previously shown to induce robust expansion of human and canine NK cells [24, 25, 29, 30]. Using this feeder line supplemented with rh-IL2 (100 IU/mL), we observed significant expansion of CD5^dim^ NK-like cells after 14–21 days in culture with a mean fold expansion of 19.0 ± 5.5 and 46.2 ± 12.7 (*P* < 0.001) at each time point, respectively (Fig. 1f). Similar to other investigators, we also observed substantial donor variation in the magnitude of NK expansion with fold-increase in NK expansion at day 14 varying from 0.6 to 67.4 (potentially related to breed variation as well as donor age). Important for the design of in vivo experiments, the kinetics of NK expansion clearly supported a log-phase of growth from day 7–14 and from day 14–21 (Fig. 1g). Finally, RT-PCR analysis confirmed a statistically significant enrichment of NK markers (NCR1) and loss of T cell markers (CD3 and CD5) in our expanded NK cells by day 14 (Fig. 1h). Taken together, these data provided evidence for successful isolation and expansion of a purified dog NK population in numbers sufficient for in vivo evaluation.

Given reports that cytokine only human NK activation techniques can lead to long-lived effector NK cells with therapeutic activity in vivo, [27, 28] we also assessed the responsiveness of non-adherent dog lymphocytes (ALAKs) to human cytokines only, including rh-IL12, rh-IL15, and rh-IL18 (Additional file 1: Figure S1). Overall, we observed the greatest expansion and cytotoxicity using a combination of rh-IL12 (50 μg/mL), rh-IL15 (50 μg/mL), and rh-IL18 (50 μg/mL) compared to other cytokine combinations. With 7 donors of diverse breeds, we observed an average of 1.8 ± 0.2 fold expansion at day 7 with rh-IL12/15/18, and cytotoxicity of these “lymphokine-activated cells” reached a peak of 60 ± 3% at 12.5:1 E:T ratio for the rh-IL12/15/18 combination (*P* < 0.0001 compared to E:T ratio of 1.6:1 and compared to individual cytokines alone, Additional file 1: Figure S1). Despite the favorable cytotoxicity of these cytokine-activated LAK cells, we elected to proceed with the feeder line-expanded and activated NK cells since the cytokine-only expansions were markedly inferior to feeder line co-culture with respect to number of effector cells generated and insufficient to permit in vivo treatment in mice or dogs.

### Expanded canine NK cells are activated and cytotoxic

**Fig. 2:**
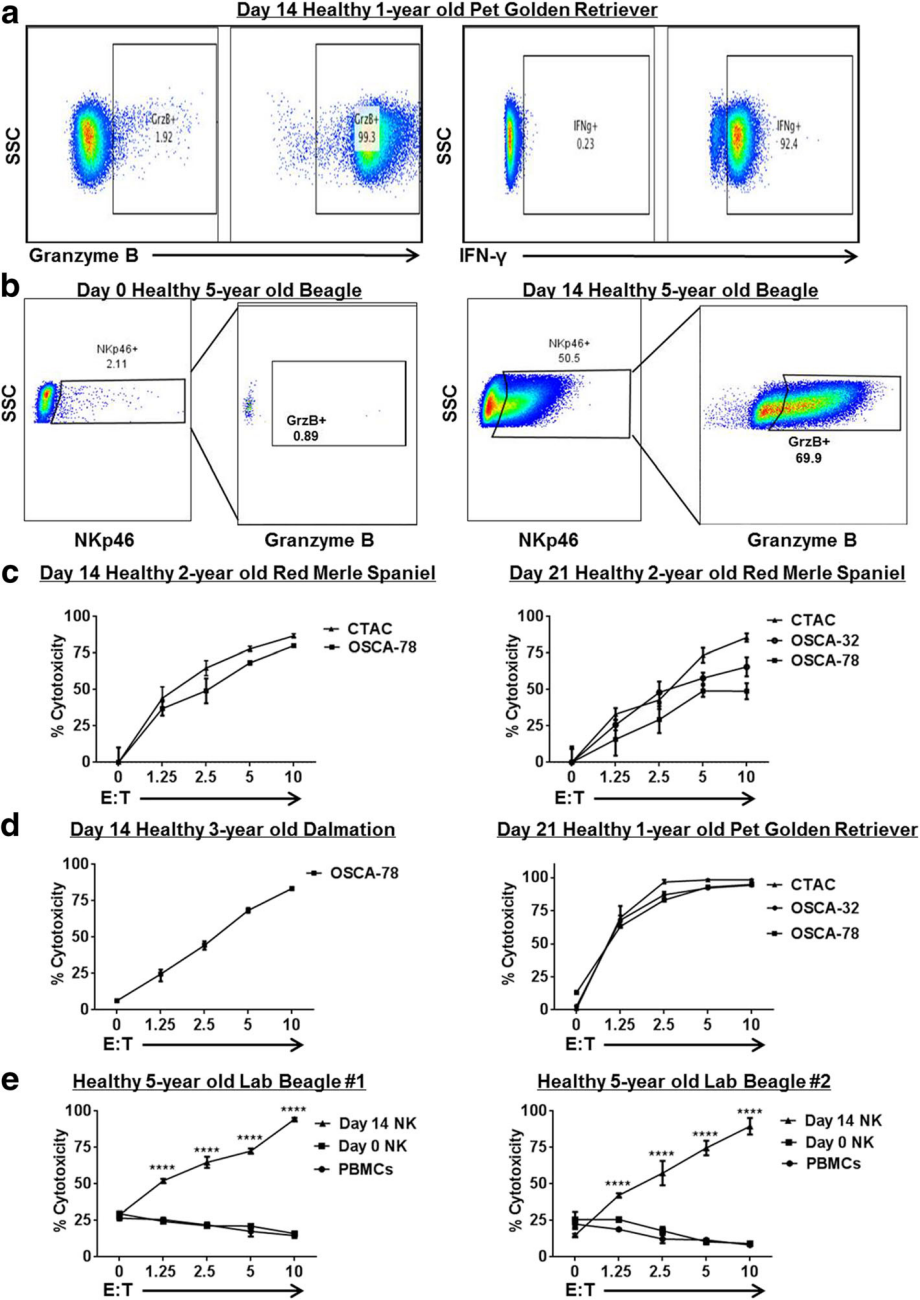
Ex Vivo Expanded Dog NK Cells are Active and Cytotoxic. Dog NK cells were CD5 depleted and co-cultured with lethally irradiated K562 Clone9.mbIL-21 and rhIL-2 (100 U/mL). After 14–21 days, NK cells were assayed by flow cytometry for expression of activation markers and cytotoxicity. **a**. Day 21 unstimulated cells show strong expression of NK activation markers Granzyme B (left) and interferon-γ (right) compared to isotype controls. In each panel, a representative experiment from a minimum of 3 replicates is shown. **b**. Post-CD5 depletion, recovered NK cells were assayed at day 0 and at day 14 for expression of NKp46 and activation marker Granzyme B. Immediately post-depletion (**Left**), there is minimal NKp46 expression with correspondingly absent Granzyme B expression in the NKp46 sub-population. At day 14 (**Right**), we observed significant upregulation of NKp46 expression with corresponding upregulation of Granzyme B. **c**. Expanded NK cells from a healthy 2-year old Red Merle Spaniel donor were assayed at day 14 (**Left**) and day 21 (**Right**) for cytotoxicity in 12–16 h killing assays with dog-specific tumor cell targets, including the dog NK sensitive CTAC tumor cell line and osteosarcoma cell lines. Dose-dependent cytotoxicity was observed. **d**. Expanded NK cells from different donors were assayed at day 14 (**Left**) and day 21 (**Right**) for cytotoxicity in 12–16 h killing assays. **e**. Blood samples were obtained at 3 time points from 2 farm beagle donors, and the cytotoxicity of fresh PBMCs (incubated with 100 IU/mL rhIL-2) was compared to CD5-depleted NK cells at day 0 (also incubated with 100 IU/mL rhIL-2) and expanded NK cells at day 14. Mean values ± SD are shown. For each panel, a representative experiment of 1–2 replicates is shown. * *P* < 0.05, ** *P* < 0.01, *** *P* < 0.001, **** *P* < 0.0001 via one-way ANOVA with Tukey’s post-test

We next assessed the functional activity of our NK cell product. After 14–21 days in co-culture with feeder cells, expanded dog NK cells were stained by flow cytometry for intracellular expression of Granzyme B and interferon-γ. Using both stimulated (phorbol 12-myristate 13-acetate 20 ng/mL and ionomycin 1 μg/mL) and unstimulated conditions, we observed >90% expression of Granzyme B and interferon-γ in our expanded cells (Fig. 2a) as well as a significant increase in the NKp46+ fraction with significant upregulation in Granzyme B expression (Fig. 2b). In addition, analysis of multiple dog donors at both 14- and 21-day time points demonstrated an increasing ability to lyse target tumor cells in vitro in a dose-dependent fashion using the canine thyroid adenocarcinoma tumor cell line, CTAC, as well as canine OSA tumor cell lines as targets (Fig. 2c **–**d). Moreover, the cytotoxicity of day 14 expanded NK cells was clearly superior to PBMCs and freshly isolated NK cells (using CD5 depletion) in a dose-dependent fashion (Fig. 2e), supporting our hypothesis that these NK cells are highly activated.

### Canine sarcoma PDX tumors are sensitive to canine NK cytotoxicity

**Fig. 3:**
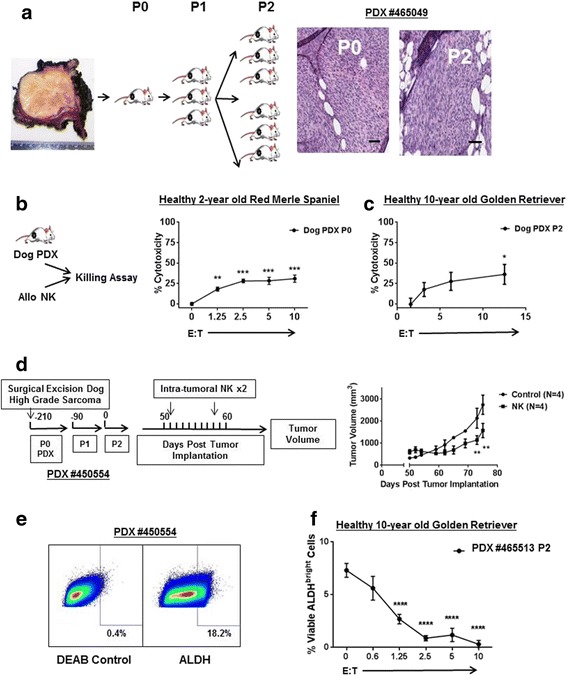
Expanded Dog NK Cells are Cytotoxic against Canine Patient-Derived Specimens including Cancer Stem Cells. **a.** The schema depicts generation of canine patient-derived xenografts from a freshly excised grade III soft tissue sarcoma. Three mm^3^ fragments were allowed to grow to approximately 15–20 mm in maximal dimension for ex vivo killing assays or for passaging into additional mice. Representative photomicrographs from passages 0 and 2 of PDX #465049 demonstrate characteristic spindle cell tumor morphology, hyperchromatic nuclei, and increased nuclear-cytoplasmic ratio consistent with canine soft tissue sarcoma which is preserved over serial passaging. Scale bar = 50 μM. **b** – **c**. When dog PDX tumors reached approximately 20 mm in size from indicated passages, they were excised and digested to create single cell suspensions. Tumor cells were then incubated with ^51^Cr and labelled cells were co-cultured with activated allogeneic dog NK cells for 4 h. Tumor cell lysis was assessed with a scintillation counter, and percent cytotoxicity was calculated using standard formulas. Representative data from 2 to 3 experiments are shown. **d**. Graphic depicts schema for in vivo experiment assessing therapeutic efficacy of intra-tumoral allogeneic dog NK cells with intra-tumoral rhIL-2 similar to our first-in-dog clinical trial protocol. P2 PDX mice received two injections one week apart of intra-tumoral NK plus 100,000 IU rhIL-2 versus control injections. Tumor volume was measured twice weekly by external caliper. **e**. A passage 2 flank PDX tumor (#450554) was excised and digested. Representative flow cytometry shows staining for diethylaminobenzaldehyde (DEAB) to control for background fluorescence and staining for ALDH^bright^ cells, representing the putative CSC sub-population. **f**. Day 14–21 activated allogeneic dog NK cells were co-cultured for 12–16 h with ex vivo digested dog PDX sarcoma tumor cells using the ALDEFLUOR system to measure the putative CSC population. * *P* < 0.05, ** *P* < 0.01, *** *P* < 0.001, **** *P* < 0.0001 via one-way ANOVA with Tukey’s post-test

We next evaluated the activity of our ex vivo expanded NK cells against dog PDX targets, including CSC sub-populations. At multiple time points (Fig. 3a **–**c), tumor tissue from canine PDX #465049 (derived from a grade III soft tissue sarcoma in a 10 year-old male mixed breed) was excised and digested to create a single cell suspension. We then co-cultured these fresh tumor specimens with day 14 or day 21 allogeneic dog NK cells at increasing E:T ratios. Similar to our in vitro results, we observed that canine NK cells from different dog donors (in separate experiments using one dog donor at a time) were able to effectively lyse sarcoma cells from freshly isolated PDX tumors in chromium release assays (Fig. 3b **-**c), reaching a maximum cytotoxicity of 30.9 ± 4.4% (*P* < 0.001 compared to non-NK controls) and 36.2 ± 12.2% (*P* < 0.01 compared to non-NK controls), respectively.

We then assessed the in vivo anti-tumor effects of adoptively transferred allogeneic dog NK cells (from a single donor) using canine PDX #450554 (derived from a surgically excised grade III fibrosarcoma in a 3 year-old female bulldog). Using passage 2 tumors measuring 5–7 mm in size, we randomized NSG mice to treatment with intra-tumoral PBS versus treatment with the combination of 100,000 IU intra-tumoral rhIL-2 and 10 × 10^6^ NK cells. Mice received 2 intra-tumoral injections 1 week apart, and the group receiving rhIL-2/NK demonstrated significant tumor growth delay (P < 0.01) which persisted until euthanasia of the control mice (Fig. 3d). Together, these data provided important pre-clinical proof-in-concept for the ability of canine NK cells to limit and reduce OSA tumor growth in vivo. Given the potential for allogeneic T cells to mediate anti-tumor effects in our PDX models, we again confirmed minimal contamination by T cells in our adoptively transferred cells (with <5–10% CD3+ cells, data not shown).

We also assessed the ability of canine NK cells to target the CSC sub-population. Similar to other investigators, we identified an ALDH^bright^ sub-population in freshly digested dog PDX sarcomas (Fig. 3e) [32]. In addition, we digested and sorted by flow cytometry a dog PDX tumor into ALDH^bright^ and ALDH^dim^ populations (Additional file 2: Figure S2) in order to validate the tumor-initiating/CSC phenotype of the ALDH^bright^ dog PDX sub-population. In agreement with our previous human data, we observed that allogeneic dog NK cells were able to effective lyse canine PDX CSCs in an ex vivo killing assay (Fig. 3f), providing data for how local NK treatment may translate to superior oncologic outcome via a CSC-targeting mechanism.

### Irradiation augments NK killing of canine sarcomas in vitro and intra-tumoral homing in vivo

**Fig. 4:**
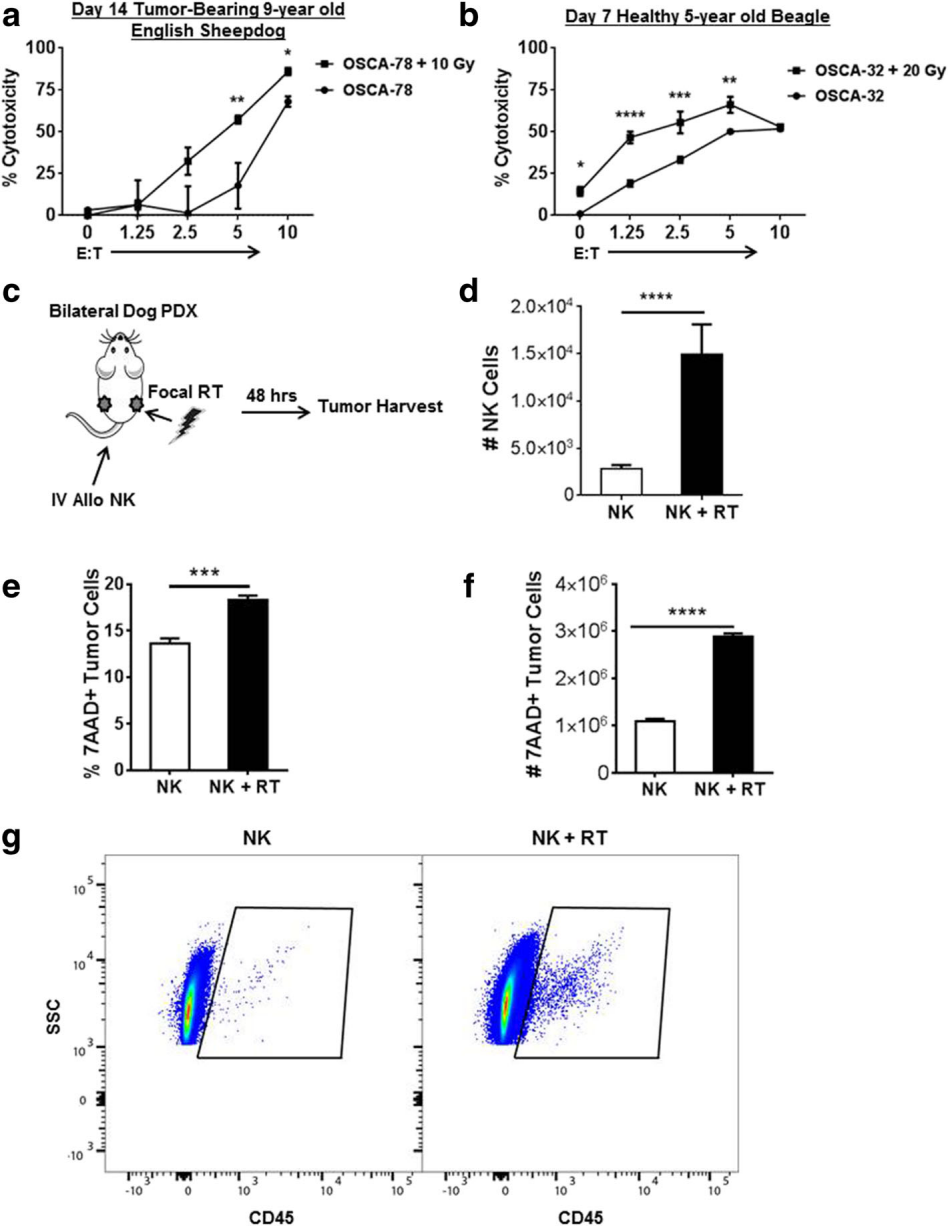
Radiation Enhances Cytotoxicity and Homing of Dog NK Cells Against Dog Osteosarcoma. **a** – **b**. Dog osteosarcoma cell lines were irradiated with 10–20 Gy. Twelve to 24 h later, irradiated cells and unirradiated controls were co-cultured with activated NK cells at increasing E:T ratios. Twelve to 16 h later, cytotoxicity was determined using dog CD45−/human CD19−/7AAD+ tumor cells to identify lysed tumor cells. NK cytotoxicity was significantly higher after RT. **c** – **d**. A single fraction of focal RT (2 Gy) was administered unilaterally to NSG mice harboring bilateral dog PDX tumors (#465049). Twelve hours later, hydrodynamic rhIL-15 was administered via tail vein followed by intravenous 14-day expanded dog NK cells. Two days later, tumors were harvested and analyzed for the presence of dog CD45+/mouse H2^d^−/7AAD- NK cells, demonstrating that significantly more NK cells trafficked to tumors after RT than to un-irradiated control tumors. Data represent one experiment with 3 mice per group. The experiment was repeated a second time with similar results. **e**. Gating on dog CD45−/ mouse H2^d^−/forward scatter-side scatter high to identify tumor cells, we observed a significantly greater fraction of the tumor cells to be non-viable after NK plus RT compared to RT alone. **f**. The higher percentage of non-viable tumor cells reflected a comparably higher number of non-viable tumor cells after NK plus RT. Representative data from two experiments are shown. **g**. Representative flow cytometry panels from panel D are shown, demonstrating significantly greater recovery of mouse H2^d^-7AAD-dogCD45+ cells in PDX tumors after RT compared to un-irradiated controls. One of 3 replicates is shown. * *P* < 0.05, ** *P* < 0.01, *** *P* < 0.001, **** *P* < 0.0001 via one-way ANOVA with Tukey’s post-test (**a**, **b**) and via paired Student’s T-test (**d** – **f**)

Since we have previously shown that RT enhances NK cytotoxicity in mouse and human solid tumor models, including sarcomas, our next objective was to assess the impact of RT on the cytotoxicity and homing of dog NK cells. After treatment with a single fraction of 10–20 Gy γ-radiation, we cultured dog OSA cells in vitro for 12–24 h before exposing them to activated allogeneic dog NK cells for 12–16 h. As shown in Fig. 4a-b, there was significantly increased NK cytotoxicity after RT.

We then assessed the effects of RT sensitization on dog NK homing and function in vivo in our canine PDX models. Using 2nd generation mice with bilateral tumors (PDX #465049 above), we administered a single fraction of 2 Gy RT using 3-D collimation to a unilateral tumor followed by i.v. injection of an rhIL-15 encoding plasmid. After 24 h, 10 × 10^6^ 21-day allogeneic dog NK cells were injected via tail vein. We harvested bilateral tumors 24 h later and analyzed for dog CD45+ mouse H2d- cells by flow cytometry (Fig. 4c). Post-RT, we recovered approximately 4X more NK cells (*P* < 0.001) in flank tumors after i.v. injection (Fig. 4d, g). In addition, we observed significantly more non-viable tumor cells in flank tumors after local RT and intravenous NK transfer (Fig. 4e-f) compared to contralateral tumors treated with intravenous NK alone, further supporting our hypothesis that RT sensitizes tumors to the cytotoxic effects of adoptively transferred allogeneic NK cells. Taken together, these data provided additional evidence in support of the synergistic anti-tumor effects of local RT with NK cells.

### Evidence for local and distant anti-tumor effects of radiation plus autologous NK in first-in-dog clinical trial

**Fig. 5:**
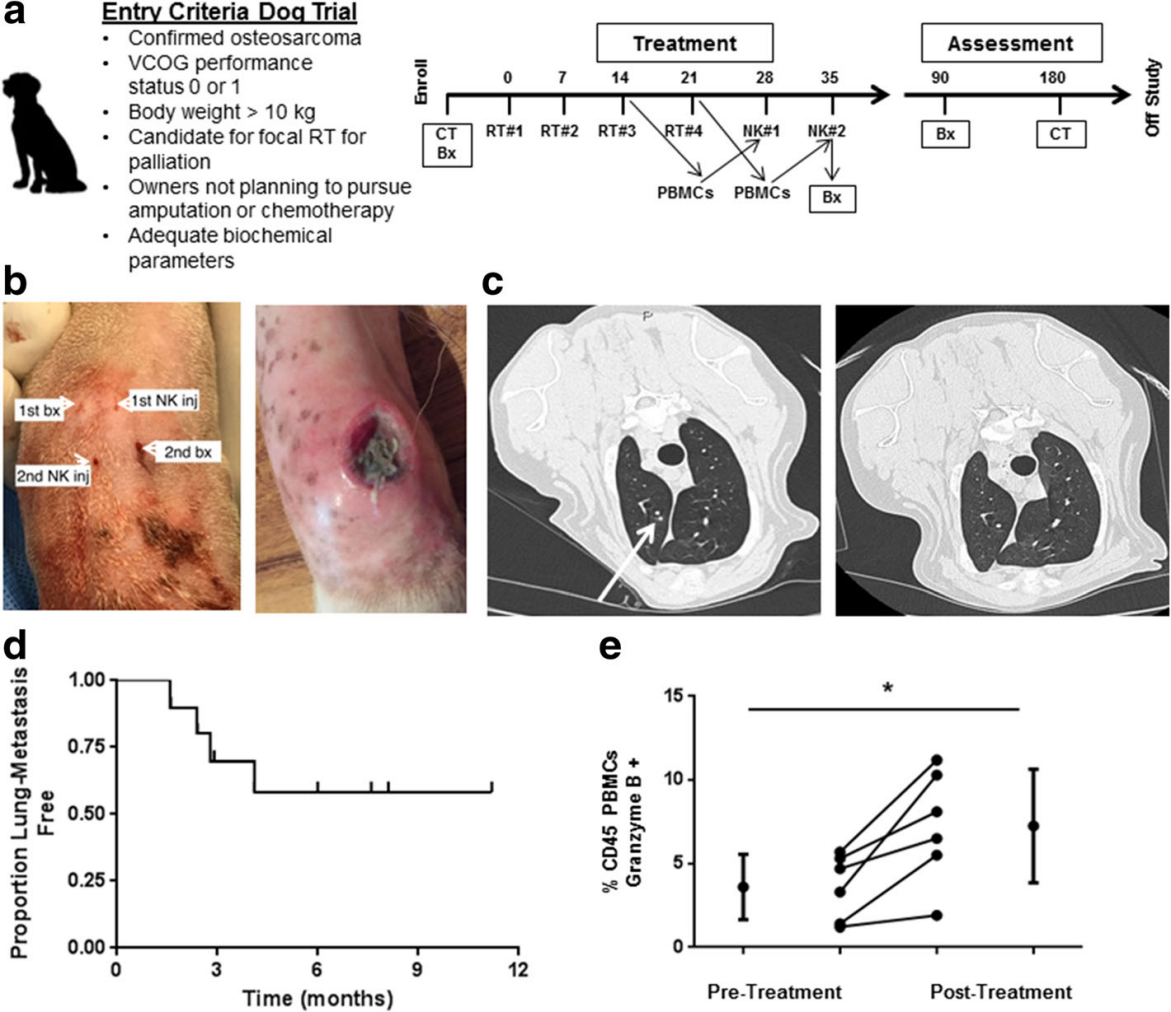
Toxicity and Outcomes in First-in-Dog Clinical Trial of Palliative RT and Intra-tumoral NK for Osteosarcoma. **a**. Graphic depicts inclusion criteria and schema for clinical trial. During the last 2 weeks of RT, peripheral blood was withdrawn for isolation, expansion, and activation of NK cells. **b**. Infection at NK injection site was observed in 3 dogs (Table 2), and management of these infections required surgical debridement in 2 dogs. (**Left**) Per protocol, we placed the needle insertion sites in distinct locations for the biopsy and NK injection procedures, and interestingly, the injection site infections occurred at the NK injection site (**Right**). **c**. Of 10 dogs with OSA treated with focal RT and autologous NK transfer who reached the 6-month primary endpoint, 5 remained metastasis-free, including resolution of suspicious pulmonary nodules in one patient. (**Left**) At 3 month follow up, chest computed tomography depicts 3 mm pulmonary nodule (arrow) that was interpreted as highly suspicious for metastatic sarcoma. At the 6-month time point, this suspicious pulmonary nodule had completely resolved (**Right**). **d**. Kaplan-Meier analysis shows the pulmonary metastasis-free survival for our clinical trial cohort. We observed a 50% proportion of lung metastases with palliative RT and autologous NK transfer (*P* < 0.05 compared to historical controls using a 1-sided test and a type I error rate of 5%). **e**. Analysis of PBMCs from our clinical trial patients demonstrated a significant increase in Granzyme B + CD45+ cells (3.6 ± 1.9% to 7.3 ± 3.4%) from baseline to post-treatment. *P < 0.05 via paired Student’s T-test

Immunotherapy ultimately needs to be evaluated in immune-competent hosts. To this end, canine tumor models offer many unique advantages as companion dogs are outbred and give rise to spontaneous tumors comparable to humans [5]. Sarcomas, including OSA, commonly afflict dogs, and canine OSA has a similar clinical course to human OSA [33, 34]. Many dogs are not candidates for (or their owners decline amputation and cytotoxic chemotherapy). Radiation of the primary tumor is frequently administered for pain relief, although lung metastases and death occur in approximately 85% of patients within 6–12 months of diagnosis [35, 36]. Therefore, as depicted in Fig. 5a, we designed a first-in-dog clinical trial for dogs with locally advanced, non-metastatic OSA whose owners were not pursuing amputation and/or chemotherapy and who were candidates for palliative RT.

The characteristics of our patient cohort are depicted in Table 1. The mean age was 7.8 years, there were 4 females, and the average weight was 50 kg. The mean number of cells injected was 1.83 ± 0.54 × 10^6^/kg and 1.67 ± 0.61 × 10^6^/kg for injections 1 and 2, respectively. Similarly, average NK viability was 90.6% ± 3.2% and 85.3% ± 9.6% for injections 1 and 2. Of 10 dogs, 5 dogs remained metastasis-free at 6 months, including one dog with resolution of a suspicious pulmonary nodule after radio-NK immunotherapy (Fig. 5b **–**c). Dogs who remained metastasis-free did receive quantitatively more injected NK cells than dogs that developed metastases, but there was significant heterogeneity among the dogs and, as a result, this difference was not statistically significant (data not shown). For our dog OSA patients treated on trial, the median follow up was 5.7 months (8.2 months for survivors), and the median lung metastasis-free survival was not reached (Fig. 5d). Similarly, progression-free survival was overall favorable compared to historical controls. As shown in Table 2, four deaths did occur secondary to metastases, while two deaths occurred secondary to other causes, including perforated ulcer (from NSAIDs) and pathologic fracture. Analysis of PBMCs pre- and post-treatment (Fig. 5e) demonstrated a significant increase in Granzyme B+ CD45+ cells (3.6 ± 1.9% vs. 7.3 ± 3.4%, *P* < 0.05).

**Table 1:** Characteristics of Patients and Expanded NK Cells for Clinical Trial

Patient	Breed	Age	Sex	Weight (kg)	Injection #1^a^ (× 10^6^)	Viability (%)	Injection #2^a^ (× 10^6^)	Viability (%)
1	Black Lab	14.8	M	29.6	30	79.3	0.3	1.0
2	St. Bernard	9.1	M	59	14.3	80.2	11.04	96.0
3	Shepherd Mix	9.1	M	23.8	392	98.9	155	100.0
4	Shepherd Mix	5.8	M	46	114.63	94.8	139.7	99.0
5	St. Bernard	1.2	F	57.6	75.93	98.6	26.7	97.2
6	St. Bernard	6.3	M	94	129.5	99.2	86.9	99.2
7	Pyrenees	7.5	F	57.1	139.5	95.0	94.5	96.0
8	Doberman	5.0	M	66	25	70.0	280	76.0
9	Retriever Mix	8.9	F	32	180	95.0	175	94.0
10	Rhodesian Ridgeback	10.2	F	35.1	115	95.0	17	95.0

^a^Expanded NK cells were documented to be LPS and Mycoplasma negative prior to adoptive transfer

**Table 2:** Treatment Outcomes Among Osteosarcoma Patients Treated in Clinical Trial of Palliative Radiotherapy and Intra-tumoral Autologous NK Cells

Patient	Age (years)	Total NK Cells Injected (× 10^6^)	Toxicity	Status	Survival Time (months)	Cause of Death^a^	6-Month Metastasis-Free Survival^b^
1	14.8	30.3		Dead	2.4	Metastases	No
2	9.1	25.34	Infection at injection site	Alive	8.1	n/a	Yes
3	9.1	547		Alive	11.2	n/a	Yes^c^
4	5.8	254.33		Alive	6.0	n/a	Yes
5	1.2	102.63	Grade 3 fever/chills/dehydration attributed to rhIL-2 reaction; Infection at injection site requiring surgery	Dead	8.1	Metastases	Yes
6	6.3	216.4	Infection at injection site requiring surgery	Dead	4.1	Perforated ulcer	No
7	7.5	234		Dead	2.9	Pathologic fracture/ metastases	No
8	5.0	305		Dead	1.6	Pathologic fracture	No
9	8.9	355		Alive	7.6	n/a	Yes
10	10.2	132		Dead	5.0	Metastases	No

^a^Animals were euthanized at owner’s request following diagnosis of index event

^b^Freedom from pulmonary metastasis 6 months after enrollment was the primary endpoint of the clinical trial

^c^Patient demonstrated resolution of suspicious pulmonary nodule post-radioimmunotherapy

**Fig. 6:**
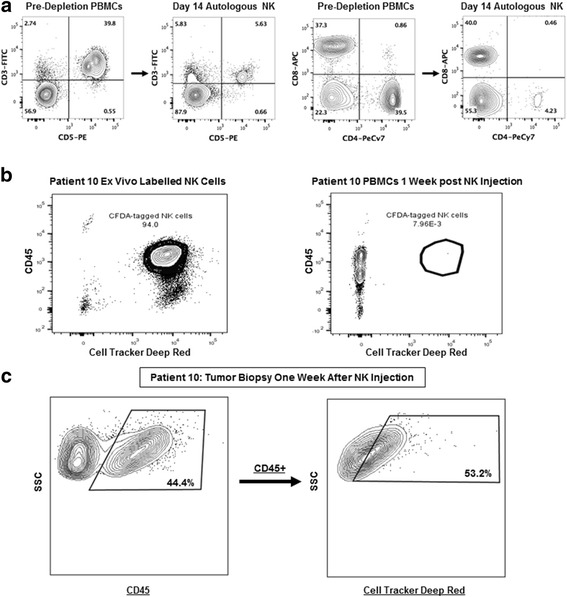
Expanded Dog NK Cells Persist in the Tumor Microenvironment post Intra-tumoral Injection. **a**. Flow cytometry pre- (**Left**) and post (**Right**)-NK isolation and expansion demonstrates a significant depletion of CD3 and CD4 cells with a residual CD3+ population after 14-days in co-culture of approximately 10% consistent with minimal T cell contamination despite the positive expression of CD8. Flow cytometry staining is shown from patient #10. **b**. Using Cell Proliferation Dye Cell Tracker Red (eFluor® 670), we labelled NK cells prior to intra-tumoral injection in patient #10. Analysis of PBMCs 1 week following NK transfer shows near uniform staining of ex vivo labelled cells (**Left**) but no evidence of recovery of labelled 7AAD-CD45 + AF647+ NK cells in the peripheral blood. **c**. In the same patient (#10) tumor biopsy 1 week following NK transfer shows recovery of (**Left**) 7AAD-CD45+ and (**Right**) labelled 7AAD-CD45 + AF647+ NK cells, indicating persistence of adoptively-transferred NK cells in the tumor microenvironment. Representative flow cytometry data of NK labelling injections from 3 patients are shown

Similar to our data in previous NK expansions, for our dog patients on the clinical trial, we observed a significant depletion of CD3 and CD4 cells after NK isolation and expansion with a residual CD3+ population of 5–10% (Fig. 6a). Based on these data, we concluded that there was minimal T cell contamination in our NK product despite the positive CD8 population since NK cells can express CD8 in multiple species [16, 19, 21]. Following Cell Proliferation Dye Cell Tracker Red (eFluor® 670) labelling of NK cells injected intra-tumorally, we recovered labelled 7AAD-CD45 + AF647+ cells from small tumor biopsies performed 1 week after intra-tumoral injection, indicating a minimum of 7-day persistence of viable NK cells injected into trial patients whereas we observed no recovery of labelled NK cells in the peripheral blood (Fig. 6b-c). These data suggest systemic activation of endogenous NK cells as a possible mechanism for the distant tumor effects we observed.

A summary of treatment toxicity is also depicted in Table 2. Overall, there was limited systemic toxicity with one case of grade 3 reaction of fever, chills, excessive salivation, and dehydration which was consistent with IL-2 toxicity. This was managed successfully by the emergency service with anti-pyretics and intravenous fluids. Additionally, we observed 3 cases of infection/ tissue breakdown at the NK injection site, including one case which required surgical debridement and surgical fixation of the underlying bone with a metal prosthesis after pathological fracture. Interestingly, as shown in Fig. 5b, 2 of the 3 cases of local infection/ tissue breakdown occurred at the NK injection site and not the biopsy site, as we endeavored to keep these needle tracts separate after the first case developed a local infection. Given the history of RT, these soft tissue infections required a prolonged period of antibiotics and other local therapies before they completely healed. It remains unresolved whether these infections were a specific reaction to our NK cell product or a non-specific inflammatory reaction secondary to anti-tumor effects and/or tissue necrosis. Despite these observed toxicities, results from our clinical trial suggest that the treatment is tolerable with clinical signals of both local and distant therapeutic effects.

## Discussion

The canine model provides a key intermediary between mouse studies and human clinical trials [5, 37]. Pet dogs are outbred animals living in the same environment as humans, and they develop spontaneous tumors over many years with an intact immune system, in marked contrast to genetically-engineered mice with accelerated tumor formation or immunosuppressed mice with implanted tumors. Additionally, canine antitumor responses can be measured using the same tools available in clinical medicine. These factors underscore the strength of the canine model for translational immunotherapy studies, and the preliminary data for efficacy which we observed for our radio-NK immunotherapy approach pre-clinically and in a first-in-dog clinical trial highlights the potential promise of this novel combination immunotherapy for dogs and humans with OSA.

Our results show that dog NK cells can be successfully isolated, activated, and expanded ex vivo and suggest that the intra-tumoral localization and cytotoxicity of these cells is significantly enhanced by pre-treatment of tumors with RT. While other investigators have characterized the phenotype and function of dog NK cells, [17, 18, 22, 24] an in vivo assessment of therapeutic effects, especially in a canine clinical trial, has not been performed. In fact, a clear strength of our work is the use of primary dog patient specimens and dog PDX samples, in addition to a dog clinical trial. These data highlight the novelty as well as the translational relevance of our work. Unlike conventional systemic administration of cytokines and adoptive cell therapy, we have adopted a strategy of intra-tumoral administration which offers theoretical advantages of reducing systemic toxicity as well as potential improved immunodulatory effects in the tumor microenvironment beyond direct cytotoxicity. Direct intra-tumoral delivery may promote broader immune effector cell cross-talk (including T-cell mediated responses) which may account for some of the systemic anti-tumor effects we observed [8, 38]. In fact, Zhang et al. observed enhanced antitumor immunity and protection against tumor rechallenge in a syngeneic model of orthotopic murine glioblastoma treated with NK cells expressing an ErbB2-specific chimeric antigen receptor [39]. Our hypothesis of greater systemic immune activation and possible bystander effects after local radio-NK immunotherapy is supported by our findings of increased Granzyme B-expressing cells in the peripheral blood post-treatment, although the extent to which this is secondary to endogenous NK cells (versus other immune effectors or cytokine administration) remains unresolved. Further studies will clarify these questions.

An additional key question to address in futures studies will be how to improve transferred NK persistence and function in vivo and to what extent transferred NK cells become hyporesponsive or exhausted in vivo. Although controversial, the role of immune checkpoints in NK cell biology is of keen interest, and greater dissection of the interaction of NK cells with checkpoint blockade may hold promise for improving NK immunotherapy and improving anti-tumor responses. The dog model is an ideal platform to evaluate these questions, especially given the recent development of a caninized monoclonal antibody to PD-1. While our data suggest that combining RT and adoptive NK transfer holds promise as a clinical strategy, it is important to acknowledge several limitations of our work. First, despite recent advances in the characterization of dog NK cells, questions still remain regarding the precise identification of these cells, including the potential for mixed T cell and NKT cell populations in our product. Recently, a canine-specific antibody to NKp46 was developed to positively identify canine NK cells. Investigators have expanded NKp46 + CD3- lymphocytes in culture in a similar method as human NK cells and demonstrated expression of several proteins that are characteristic of human and mouse NK cells such as CD16, NKp30, and DNAM-1. Interestingly, our data show that the NKp46 + CD3- cells were also CD5^dim^, offering important confirmatory evidence that earlier characterizations of canine NK cells were, in fact, correct. Despite the moderate expression of CD8, our results suggest that our CD5 depletion strategy successfully eliminates T cells and thereby enriches for NKp46 + CD3- NK cells. Our data suggests that CD3 expression is an indicator of poorly depleted T cells (similar to humans), but that dog NK cells can display robust CD8 expression in approximately 40% of cells. Although we did observe significant enrichment of NKp46 + CD3- cells during ex vivo expansion, we recognize that approximately 50% or more of our “NK” product remained NKp46-. Although the large majority of these cells were Granzyme B+ (and therefore may represent NKp46- or “null” NK cells), [24] it is important to acknowledge the implication of these results, including the likely presence of NK sub-populations and/or mixed cell populations in these expansions which remain a source for heterogeneity in current and future results. We also observed heterogeneity in the magnitude of our NK expansions (while robust cytotoxicity remained fairly reproducible). We suspect that breed and age differences in our dog subjects played a significant role in these results, and further research will be needed to elucidate these potentially important differences.

Although we did observe evidence of therapeutic effects, including apparent abscopal effects in our first-in-dog clinical trial, it is reasonable to hypothesize that intra-tumoral NK therapy using autologous NK cells may have limited efficacy in treatment of systemic disease, especially when tumor burden and extent of metastases are greater. Prior to adoptive transfer, our data demonstrate that our NK cell product is maximally activated in culture, but the in vivo environment (post transfer) can lead to NK anergy and hypo-responsiveness when co-stimulation and cytokine support are decreased. In our clinical trial, we chose to utilize autologous NK transfer because of the decreased potential toxicity and greater engraftment with autologous NK cells compared to allogeneic NK cells. Although RT appears to overcome some of the drawbacks of autologous NK therapy, additional studies are needed to address the advantages and disadvantages of autologous vs. allogeneic NK transfer in canine models and to explore mechanistic approaches to optimize this therapy in dogs and eventually humans. Similarly, because of limited characterization of inhibitory KIR-DLA interactions in dogs, we are unable to assess the impact of these receptor-ligand interactions in our models at this time. Another question to address is the mechanism of killing with our expanded dog NK cells. Although we observed a trend for greater target cell killing in long term (16 h) NK killing assays, we did not perform blocking experiments using antagonists to FasL or TRAIL to more rigorously evaluate NK killing mechanisms. Therefore, more formal studies are needed to determine whether dog NK cells kill preferentially by perforin-granzyme or death receptor pathways, particularly since expression of death receptor ligands is variable among tumor cells in vivo and may therefore impact efficacy.

Finally, another factor in our studies which may confound our results is the use of xenogeneic cytokines, especially in our dog clinical trial. Numerous studies have shown that recombinant human cytokines are active in non-human primates, dogs, and other mammals, [40–43] reinforcing that human cytokines are feasible for proof-in-concept studies in dogs. However, neutralizing antibody formation has been observed with the potential for anaphylaxis with longer courses of treatment. Since Foltz et al. observed greater purity and responsiveness of canine NK cells after stimulation with canine IL-2 versus human IL-2, the use of clinical grade canine cytokines will represent a key variable in future studies. Moreover, there is growing consensus that IL-15 is superior to IL-2 to support NK cells activity in vivo. In our clinical trial, we used intra-tumoral injection of rhIL-2 to achieve in vivo cytokine support of our adoptively-transferred dog NK cells since previous studies have shown rhIL-2 to be well tolerated in dogs. For our first-in-dog clinical trial of adoptively transferred NK cells, we were reluctant to add the uncertainty (as well as potential for toxicity) of rhIL-15 which has never been administered in dogs. Yet, despite these limitations, clinical trials in dogs provide a key resource to answer questions about toxicity and efficacy and thereby speed clinical translation.

## Conclusions

In summary, we are able to show that RT enhances homing and cytotoxicity of dog NK cells in pre-clinical models of dog sarcomas, including clinically meaningful dog PDX tumors. We also report the first-in-dog clinical trial of adoptive transfer of intra-tumoral NK cells with promising results. If, as we hypothesize, the sensitizing effects of RT can be applied more broadly prior to the application of NK cell therapy, then the therapeutic impact of NK cells both locally and distantly can be amplified, potentially leading to greater and more sustained treatment effects in vivo for OSA and other refractory malignancies in dogs and people.

## Additional files

Additional file 1: Figure S1. Canine Lymphokine Activated Killer Cells Respond to Human Cytokines and Can Target Dog Osteosarcoma Cells. Dog PBMCs were obtained from healthy dogs and laboratory beagles. Adherent lymphocytes were isolated by standard techniques and cultured with short term rhIL-12/15/18 for 24 h followed by co-culture with low dose rhIL-2 (100 IU/mL) for 7 days. Cells were assessed for expansion, viability, and cytotoxicity at various time points. A. From 4 donors, the mean number of ALAKs at day 0 was 12 × 10^6^ ALAKs. After 7 days in culture, the mean number of recovered ALAKs was 23 ± 9.8 × 10^6^ cells. B. After 7 days in culture, the mean fold expansion of ALAKs was 1.8 ± 0.3. C. Mean viability decreased from 97.7 ± 1.8% on day 0 to 92.3 ± 4.7% on day 7. D. Using PBMCs from a 4-year old healthy unknown breed, we observed that cytotoxicity against OSA-1 targets at day 7 was significantly greater after co-culture with recombinant human cytokines IL-12 (10 ng/mL), IL-15 (10 ng/mL), and IL-18 (10 ng/mL) compared to rhIL-2 alone (5000 IU/mL). E. Using ALAKS expanded with rhIL-12/15/18 from a healthy 7-year old Rat Terrier, we performed a 12–16 h killing assay at the indicated effector:target ratios with OSCA-32. Dose-dependent cytotoxicity was again observed. **** *P* < 0.0001 via one-way ANOVA with Tukey’s post-test. (TIFF 104 kb)

Additional file 2: Figure S2. Validation of ALDH as a CSC Marker in Dog PDX Tumors. A. A dog sarcoma PDX tumor was allowed to grow to ~ 20 mm in maximal dimension. The tumor was then excised and digested into single cell suspension. B. Tumor cells were sorted by flow cytometry into ALDH^bright^ and ALDH^dim^ populations. 2 × 10^5^ purified cells were implanted subcutaneously into contralateral flanks of NSG mice (*N* = 4) and allowed to grow. ALDH^bright^ cells established tumors faster and were more rapidly fatal. * *P* < 0.05 via one-way ANOVA with Tukey’s post-test. C. Representative photograph showing difference in tumor formation between ALDH^bright^ and ALDH^dim^ sarcoma PDX #465049 cells implanted subcutaneously in NSG mice. (TIFF 890 kb)

## Abbreviations

ALAKs: Adherent lymphokine-activated killer cells
ALDH: Aldehyde dehydrogenase
CSC: Cancer stem cell
CT: Computed tomography
E:T: Effector-to-target
IL: Interleukin
NK: Natural killer
OSA: Osteosarcoma
PBMCs: Peripheral blood mononuclear cells
PBS: Phosphate-buffered saline
PDX: Patient-derived xenograft
RH: Recombinant human
RT: Radiation
SQ: Subcutaneous
