# Supplementary figures and images for: Radiotherapy enhances natural killer cell cytotoxicity and localization in pre-clinical canine sarcomas and first-in-dog clinical trial
Source: J Immunother Cancer. 2017 Dec 19;5:98. doi: 10.1186/s40425-017-0305-7 (PMC5735903; doi:10.1186/s40425-017-0305-7)

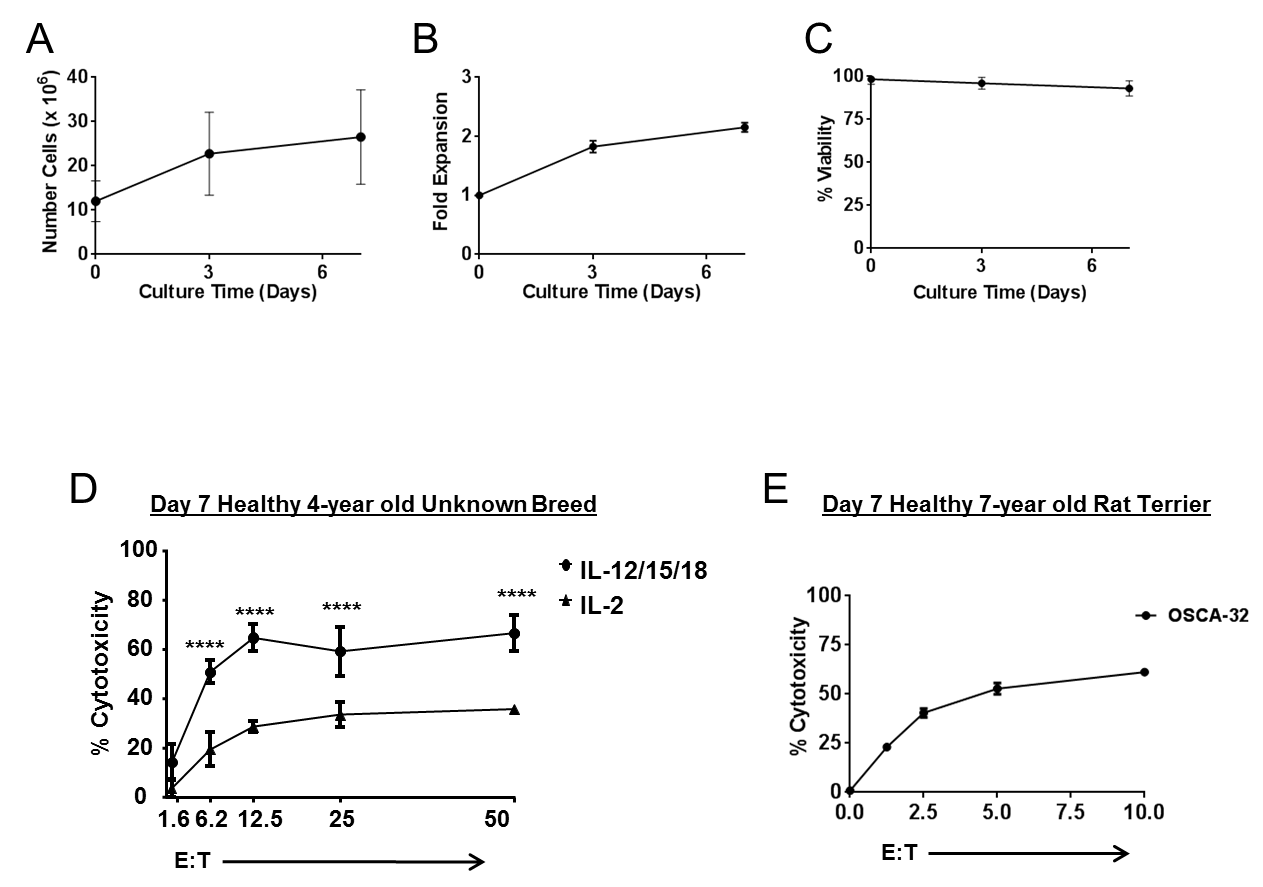

Supplement: Supplementary file 1 — Canine Lymphokine Activated Killer Cells Respond to Human Cytokines and Can Target Dog Osteosarcoma Cells. Dog PBMCs were obtained from healthy dogs and laboratory beagles. Adherent lymphocytes were isolated by standard techniques and cultured with short term rhIL-12/15/18 for 24 h followed by co-culture with low dose rhIL-2 (100 IU/mL) for 7 days. Cells were assessed for expansion, viability, and cytotoxicity at various time points. A. From 4 donors, the mean number of ALAKs at day 0 was 12 × 106 ALAKs. After 7 days in culture, the mean number of recovered ALAKs was 23 ± 9.8 × 10^6^ cells. B. After 7 days in culture, the mean fold expansion of ALAKs was 1.8 ± 0.3. C. Mean viability decreased from 97.7 ± 1.8% on day 0 to 92.3 ± 4.7% on day 7. D. Using PBMCs from a 4-year old healthy unknown breed, we observed that cytotoxicity against OSA-1 targets at day 7 was significantly greater after co-culture with recombinant human cytokines IL-12 (10 ng/mL), IL-15 (10 ng/mL), and IL-18 (10 ng/mL) compared to rhIL-2 alone (5000 IU/mL). E. Using ALAKS expanded with rhIL-12/15/18 from a healthy 7-year old Rat Terrier, we performed a 12–16 h killing assay at the indicated effector:target ratios with OSCA-32. Dose-dependent cytotoxicity was again observed. **** P < 0.0001 via one-way ANOVA with Tukey’s post-test. (TIFF 104 kb) [file 40425_2017_305_MOESM1_ESM.tif]

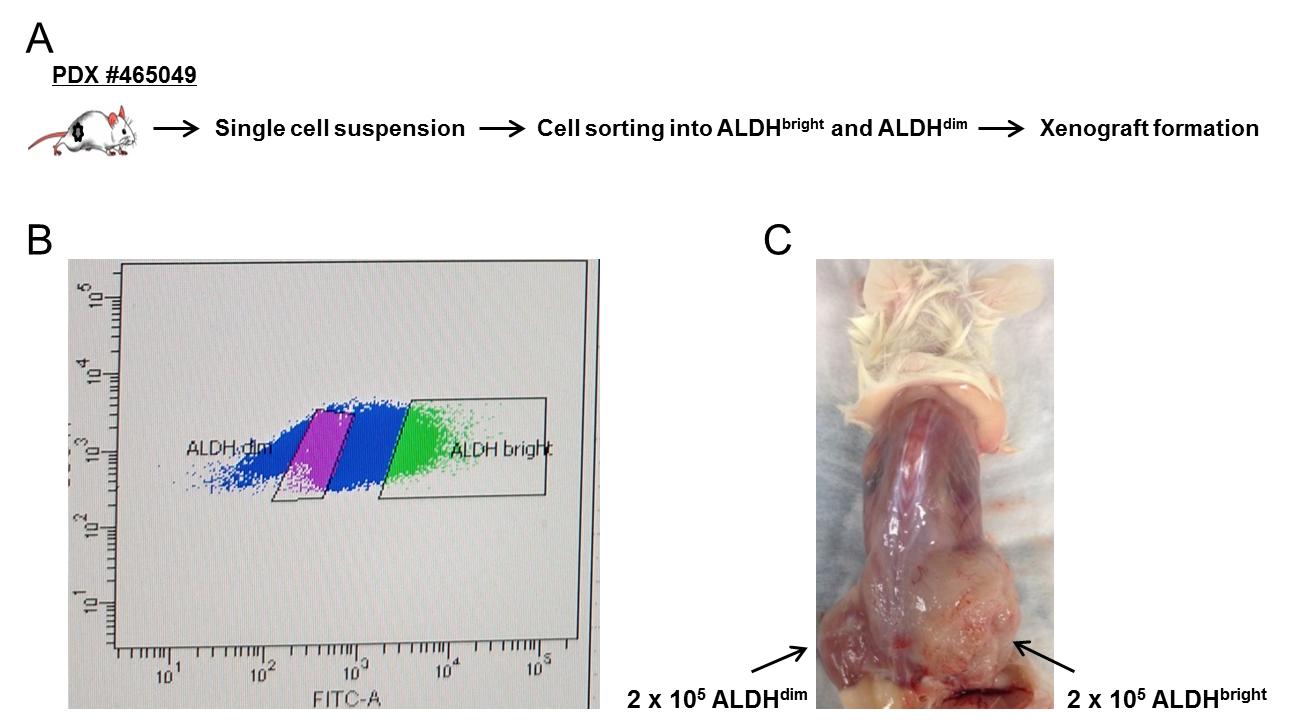

Supplement: Supplementary file 2 — Validation of ALDH as a CSC Marker in Dog PDX Tumors. A. A dog sarcoma PDX tumor was allowed to grow to ~ 20 mm in maximal dimension. The tumor was then excised and digested into single cell suspension. B. Tumor cells were sorted by flow cytometry into ALDHbright and ALDHdim populations. 2 × 10^5^ purified cells were implanted subcutaneously into contralateral flanks of NSG mice (N = 4) and allowed to grow. ALDHbright cells established tumors faster and were more rapidly fatal. * P < 0.05 via one-way ANOVA with Tukey’s post-test. C. Representative photograph showing difference in tumor formation between ALDHbright and ALDHdim sarcoma PDX #465049 cells implanted subcutaneously in NSG mice. (TIFF 890 kb) [file 40425_2017_305_MOESM2_ESM.tif]
